# Collective guiding of acoustically propelled nano- and microparticles

**DOI:** 10.1039/d2na00007e

**Published:** 2022-05-14

**Authors:** Tobias Nitschke, Joakim Stenhammar, Raphael Wittkowski

**Affiliations:** Institut für Theoretische Physik, Center for Soft Nanoscience, Westfälische Wilhelms-Universität Münster 48149 Münster Germany raphael.wittkowski@uni-muenster.de; Division of Physical Chemistry, Lund University SE-221 00 Lund Sweden

## Abstract

One of the most important potential applications of motile nano- and microdevices is targeted drug delivery. To realize this, biocompatible particles that can be guided collectively towards a target inside a patient's body are required. Acoustically propelled nano- and microparticles constitute a promising candidate for such biocompatible, artificial motile particles. The main remaining obstacle to targeted drug delivery by motile nano- and microdevices is to also achieve a reliable and biocompatible method for guiding them collectively to their target. Here, we propose such a method. As we confirm by computer simulations, it allows for the remote guiding of large numbers of acoustically propelled particles to a prescribed target by combining a space- and time-dependent acoustic field and a time-dependent magnetic field. The method works without detailed knowledge about the particle positions and for arbitrary initial particle distributions. With these features, it paves the way for the future application of motile particles as vehicles for targeted drug delivery in nanomedicine.

## Abstract 2

A new method for efficiently guiding motile nano- and microparticles to a target in a complex environment such as the human vasculature is proposed and computationally demonstrated, with potential applications in targeted drug delivery.

## Graphical abstract

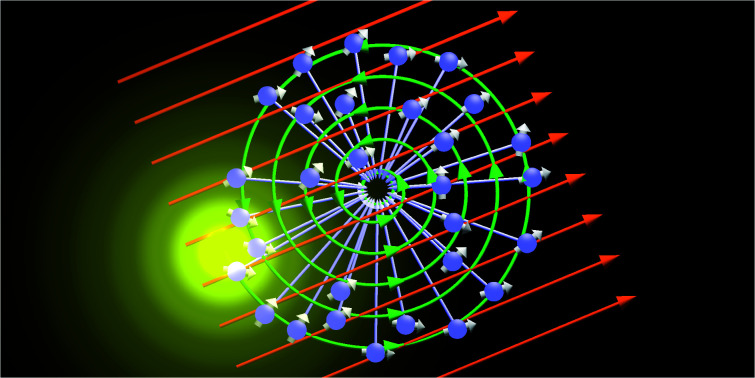

## I. Introduction

The individual and collective properties of motile nano- and microdevices, also called “active particles”,^1^ form one of the most fascinating and fastest evolving areas of nanotechnology.^2–19^ Benefiting from earlier progress in the fabrication of nanoparticles, research of the last two decades has resulted in a large number of realizations of artificial nano- and microparticles with different propulsion mechanisms.^1,2,4,9,11,12,15,16,19^

An important example is acoustically propelled particles.^16,19,20^ These particles typically have a polar shape and move when they are exposed to ultrasound due to unbalanced hydrodynamic stresses that a surrounding liquid exerts onto their surface. In contrast to other propulsion mechanisms, the acoustic propulsion works for a wide range of particle shapes,^20–24^ sizes,^20,21^ and materials,^20,25,26^ as well as surrounding liquids.^27–30^ However, the understanding of the influence of these properties on the acoustic propulsion is very limited. The few known requirements for these properties include that the particle shape or the distribution of the mass density should have a preferential direction,^22,26,31^ that the particle size or ultrasound intensity should be sufficiently large to ensure that the acoustic propulsion dominates Brownian motion,^23^ and that a higher mass density of the particle material compared to the surrounding liquid increases the propulsion speed.^20,25^ Another advantage of acoustic propulsion compared to other (*e.g.*, chemical) propulsion is that the particles can persistently be supplied with energy *via* the ultrasound and do not run out of fuel after some time.

Being intrinsically far from thermodynamic equilibrium, motile nano- and microparticles are highly interesting from a nonequilibrium statistical physics perspective,^32^ while a somewhat less developed driver for research on ultrasound-propelled and other active particles is their plethora of important potential applications. Among them are applications in environmental protection,^8,10,13,17,33^ materials science,^1,34–36^ and medicine.^5,9,29,37–43^ Particularly great attention is payed to the area of drug delivery,^3,8,44^ where motile nano- and microparticles can enable a fast local distribution of drugs^3,8,37,41,44,45^ or their targeted delivery to specific sites.^3,8,21,26,27,39,40,42–44,46^ While enhanced local drug distribution has already been demonstrated *in vivo*,^3–6,10,37,41,45^ directed delivery of therapeutic and imaging agents to a distant target remains a big challenge.^3,7,9–11,15^

For a realization of targeted drug delivery by motile nano- or microparticles, five basic requirements have to be met:^4,6,7,9–12,15,47^

(1) Biocompatibility of the particles and their propulsion mechanism.^4–12,44,47,48^ In particular, the particles must not consist of a toxic material or be propelled by a toxic fuel. To exclude negative long-term effects of the particles, they should also be removable or biodegradable.^3–5,7,9,10,12,14,15,44,47,48^

(2) The ability of the particles to move actively, with sufficient speed, and over sufficiently long periods of time inside the body of a patient.^10,11,15,48^ This excludes, *e.g.*, particles that need to be illuminated for propulsion.^17,49^

(3) The particles must allow for the encapsulation and release of drug molecules.^7,12,46^

(4) It should be possible to functionalize the particles^9,10,14^ and to equip them with stealth features to go undetected by the immune system.^4,5,11,12,14^

(5) A robust and reliable method that allows for steering the particles to their target.^7,9–12,15,47,48^

Fortunately, most of these requirements can already be fulfilled by using current technologies. The first problem can be solved by using ultrasound-propelled nano- or microparticles. Since this propulsion mechanism works for a wide range of particle materials, this can be chosen to ensure biocompatibility^12,26,28,30,50^ and biodegradability.^3,19^ Furthermore, the acoustic propulsion mechanism itself is biocompatible.^9,16,19,26,27^ Ultrasound propulsion can yield considerable particle speeds already for ultrasound intensities that are typically used in sonography^22,23,31^ and are harmless for patients. Such particles also fulfill the second requirement, since acoustic propulsion works in various liquids including biofluids^27,28,30^ and since the particles can be supplied with energy as long as necessary *via* the ultrasound. The third requirement can be fulfilled by combining the particles with established techniques for encapsulation and release of drugs that are widely used in the context of nanocarriers.^12,50–52^ Since the acoustic propulsion mechanism does not rely on a particular particle material, it is furthermore possible to use established techniques for functionalization of nanoparticles^27,30,50^ and equipping them with stealth features^11,49^ to fulfill the fourth requirement.

Hence, the main remaining requirement is a method that allows for guiding acoustically propelled nano- and microparticles to a particular target.^9–11,15^ To be relevant for medical applications, this method must be biocompatible and reliable.^7,12^ Furthermore, it must work for large numbers of particles^10^ with arbitrary (and unknown) initial distributions. It must also be robust over large distances^9^ and in complex environments with unknown structure, such as the vasculature of a patient.^15^ Finally, it should not rely on tracking of the particle positions or orientations. Previous experimental studies have considered acoustically propelled particles that are steered by controlling their orientation using an external magnetic field.^21,26,27,29,50^ By observing the particles through a microscope and adjusting the orientation of the magnetic field (and thus of the particles) depending on their current position and a path along which they are supposed to move, it is possible to guide them along prescribed paths. However, this method requires tracking of the particles' positions in real time with a microscope, which is not possible in medical applications. Moreover, this method works only for single particles. When several particles need to be steered at the same time, like in a drug-delivery application,^4,7,10,15^ the orientations of all particles are manipulated in the same way so that all particles move along similar trajectories with offsets that originate from their different initial positions, which prevents guiding all particles towards a common target. In a typical application, particles would furthermore distribute through a combination of convection by the blood flow and their propulsion in the vasculature surrounding the target. Applying the presently existing guiding method would then in principle allow for the guiding of one particle precisely to the target, while other particles, especially if they start on the opposite side of the target, would move away from it. A guiding method for medical applications needs to overcome these limitations and allow for collective guiding to a common target without the need for particle tracking.

In this article, we propose a method for collective guiding of acoustically propelled nano- and microparticles that meets all the aforementioned criteria. The proposed method combines a space- and time-dependent ultrasound field that propels the particles with a time-dependent magnetic field that collectively aligns their propulsion directions. Using computer simulations, we demonstrate the feasibility of using this guiding method for potential applications in nanomedicine.

## II. Results and discussion

A method for guiding motile nano- and microparticles should be as simple as possible to facilitate its application. Therefore, we do not pursue ideas to equip the particles with data processing units that control the propulsion of the particles and guide them to the target,^53^ whose large-scale fabrication would be extremely challenging and expensive. Instead of such an internal guiding of the particles, we use a method that is based on external guiding. This means that external fields are applied to influence the motion of the particles in such a way that they collectively move towards the common target. For reasons of efficiency, the external fields should not simply pull or push the particles towards the target, but rather make use of the particles' propulsion to guide them in the right direction.

In principle, several types of external fields could be used to influence the motion of the particles. However, we focus only on acoustic and magnetic fields since it is possible to generate and control these fields, they run without significant absorption through biological tissue, and they are harmless for a patient if the intensity and frequency of the acoustic field and the flux density and temporal variation of the magnetic field are sufficiently low. By taking both fields into account simultaneously, we increase the number of available degrees of freedom compared to using only one of them, and this allows us to achieve a high degree of control using relatively simple fields, as discussed below. Furthermore, since both fields can in principle be space- and time-dependent, they provide a versatile method to control the particle motion.

The simplicity of the field structure is a particularly important point, since it simplifies the experimental realization. In particular, using only a magnetic field could require the generation of a set of field lines that is not divergence-free and thus not possible to generate. It also needs to be taken into account that the fields need to be realized with realistic tools like a phased array transducer and magnetic field coils placed outside of the patient. Since acoustic fields are easier to structure spatially than magnetic fields, it is reasonable to use the spatial degrees of freedom of the acoustic field and to keep the structure of the magnetic field simple. Furthermore, the time dependence of the acoustic field can be large, whereas the magnetic field should change slowly in order to avoid effects of electromagnetic induction.

Based on these considerations, we now propose a method for guiding ultrasound-propelled particles. It is based on the combination of a space- and time-dependent ultrasound field, a time-dependent magnetic field, and magnetic particles. Making particles magnetic is typically possible by embedding a single solid magnetic core or a number of smaller magnetic beads^43^ into the particle or by coating it with a magnetic shell. By using a suitable magnetic material such as magnetite, it is furthermore possible to retain biocompatibility of the particles. When the magnetic particles are ferro- or ferrimagnetic, their orientation can be controlled by a homogeneous external magnetic field. For (super)paramagnetic particles, an inhomogeneous magnetic field can be used to control the particle orientation.

The main idea of our method is to use a focused ultrasound beam to locally supply the particles with energy as well as a magnetic field to control their orientation. Since the propulsion speed increases with the ultrasound intensity,^23^ only those particles that are within the focus of the beam are strongly propelled, whereas the other particles in the system are only weakly propelled or not at all. The magnetic field is then oriented so that it points from the focus towards the target, causing the fast particles to move towards the target,^54^ while the motion of the remaining particles is negligible due to their low propulsion speed. The focus of the ultrasound beam moves continuously through the system, while the magnetic field rotates with it so that it always points along the vector from the focus to the target. This makes the motile particles move towards the target, whereas for the out-of-focus (immotile) particles, only the orientation is changed. A possible trajectory for the focus is a spiral that starts in the outer regions of the system and ends with a cyclic motion along the surface of the target. This trajectory helps keeping the temporal variation of the magnetic field slow and thus to avoid effects of electromagnetic induction. With this combination of two fields, the particles of the whole system move towards and collectively accumulate in the target, independent of their initial positions and orientations. The method furthermore does not require any knowledge of the initial particle distribution or the particle positions and orientations during the course of the trajectory.

This method fulfills all criteria mentioned in Section I, with the nice additional feature that the target does not need to have a particular size or shape.

**Fig. 1:**
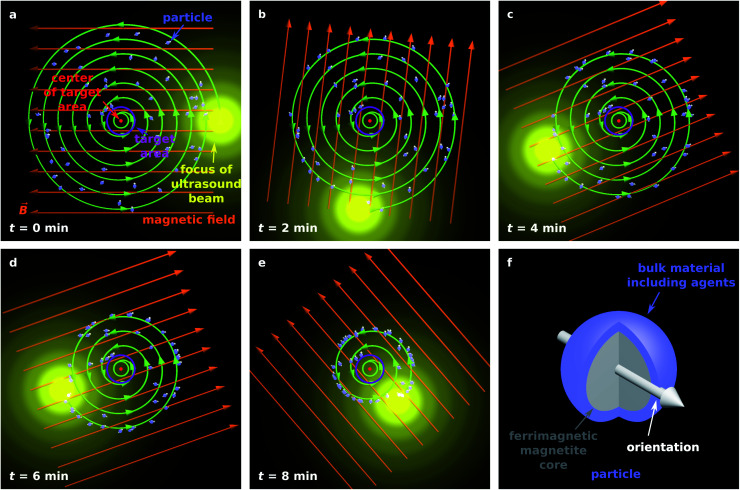
(a–e) Illustration of the proposed particle guiding method. (f) Illustration of a single particle suitable for targeted drug delivery.

Fig. 1a–e illustrates the proposed guiding method for 50 magnetic particles in the *x*–*y* plane.

Although acoustically propelled particles can have various shapes,^20,21,55^ our model for convenience assumes spherical particles. We start from a random, homogeneous initial particle distribution within a disk-shaped region (Fig. 1a) with a circular target area at its center. The ultrasound beam is taken to be antiparallel to the *z*-axis, and its focus moves in the *x*–*y* plane along a spiral towards the target, while a homogeneous external magnetic field with flux density *B⃑* rotates within the plane so that it is always parallel to the vector from the center of the focus of the ultrasound beam to the center of the target. Note that Fig. 1a shows the initial particle orientation, where the particles are not yet aligned with the magnetic field. At later times (Fig. 1b–e), the mean orientation of the particles is aligned with the magnetic field, but due to Brownian rotation the orientations of the individual particles can deviate from the orientation prescribed by the magnetic field. In Fig. 1f, a schematic example of a magnetic particle is shown, composed of a ferrimagnetic magnetite core and a shell of a bulk material with embedded therapeutic agents, similar to the particle design in ref. 43.

In the following, we numerically demonstrate the reliability and robustness of the proposed method. To this end, we performed particle-based Brownian dynamics simulations of a system of magnetic ultrasound-propelled particles being guided towards a common target using the method described above. Further details on the computational implementation are given in the Methods section.

**Fig. 2:**
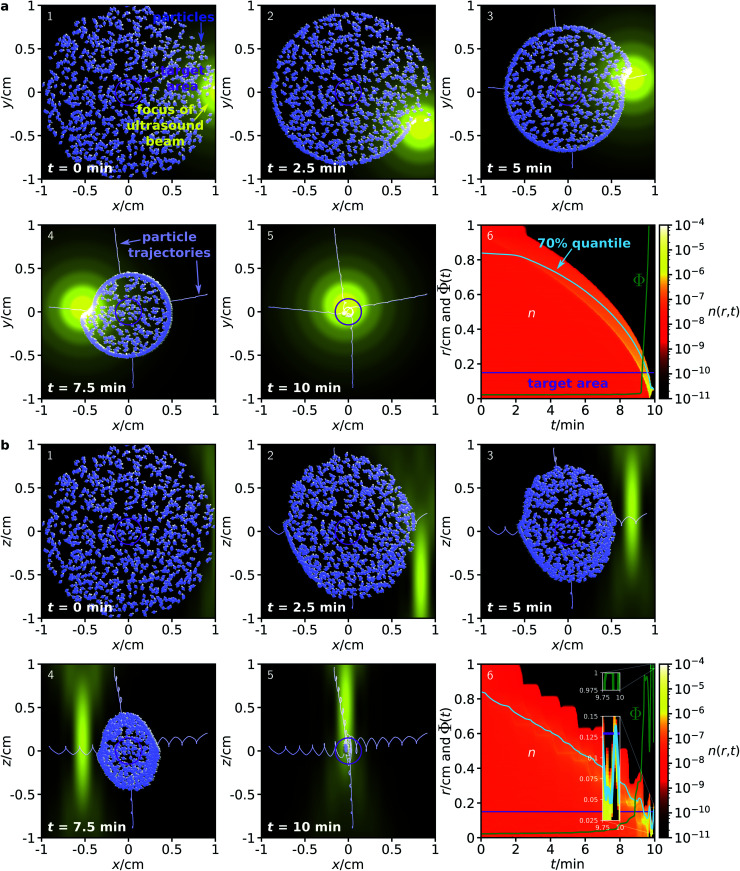
Time evolution of a distribution of *N*_p_ = 1000 particles with diameter *σ* = 100 nm in the (a) *x*–*y* plane and (b) *x*–*z* plane with a homogeneous environment. Panels 1–5 show the particle distribution for increasing times, where the particles are enlarged by a factor 2000 to be visible. For 4 representative particles, also their trajectories are shown. Panels 6 show the radial particle distribution *n*(*r*,*t*) denoting the mean packing density at a distance *r* from the target (bin width 50 μm) at time *t*, the corresponding 70% quantile, the boundary of the target area, and the fraction of particles *Φ*(*t*) that are within the target at time *t*, ensemble averaged for 100 simulations.

First, we consider a system where the particles are guided through a homogeneous environment.^40^ The system is similar to that shown in Fig. 1, but now it contains *N*_p_ = 1000 particles with diameter 100 nm that are initially distributed in a disk of diameter 2 cm, and guided towards a target area with a diameter of 3 mm. Fig. 2 shows the time evolution of the particle distribution obtained from our simulations.

To demonstrate that the method allows for full, 3-dimensional control of the particle motion, we now consider two separate initial particle distributions: one in the *x*–*y* plane (Fig. 2a) and one in the *x*–*z* plane (Fig. 2b). To achieve particle guiding in a 3-dimensional system, one can either subsequently perform the procedures for particle guiding in respectively the *x*–*y* plane and the *x*–*z* plane, or combine them in different ways (*e.g.*, by choosing a sequence of spherical spirals with decreasing radius for the trajectory of the focus). In both procedures, the ultrasound beam remains antiparallel to the *z*-axis, while its focus can move within the corresponding plane. Furthermore, the magnetic field can rotate within the plane. This could be realized in medicine with a single phased array transducer on one side of a patient and with three pairwise perpendicular pairs of static magnetic field coils with tunable flux densities or one pair of field coils that can rotate around its center. As is apparent from Fig. 2, the proposed method works very well for both initial conditions, although the focus of the ultrasound beam has very different profiles in the two cross sections. Within 10 minutes, all particles are guided into the target area. Remarkably, this method works independently of the position or distance of the particles relative to the target and without any knowledge of the particle positions or orientations during the guiding procedure. Comparing the size of the initial particle distribution with the small particle size, some of the particles are guided over very long distances to the target.

Although we do not provide a detailed analysis on how the flux density and rate of rotation of the magnetic field influences the guiding performance, we can assess the qualitative effects: When the magnetic field is much weaker, it is no longer able to align the particles and the guiding method will not work. For much stronger magnetic fields, the guiding method should work even better, but the magnetic fields can become harmful for a patient due to increased electromagnetic induction. When the rate of rotation is lower, the particle guiding should work better, but the guiding of the particles to the target will be slower. Faster rotating magnetic fields will increase electromagnetic induction to potentially harmful levels.

Next, we consider a system where the particles are steered through a complex environment, loosely inspired by the structure of a vascular system. To test the proposed method for the case of a complex environment, we performed simulations where particles are confined within a network of channels, having a constant diameter of 500 μm. This channel system is only a simple model network and not equivalent to a real human vasculature, but sufficient to test whether the proposed guiding method can work also for particles that are confined in a channel system. The simulations are otherwise analogous to those for a homogeneous environment described above.

**Fig. 3:**
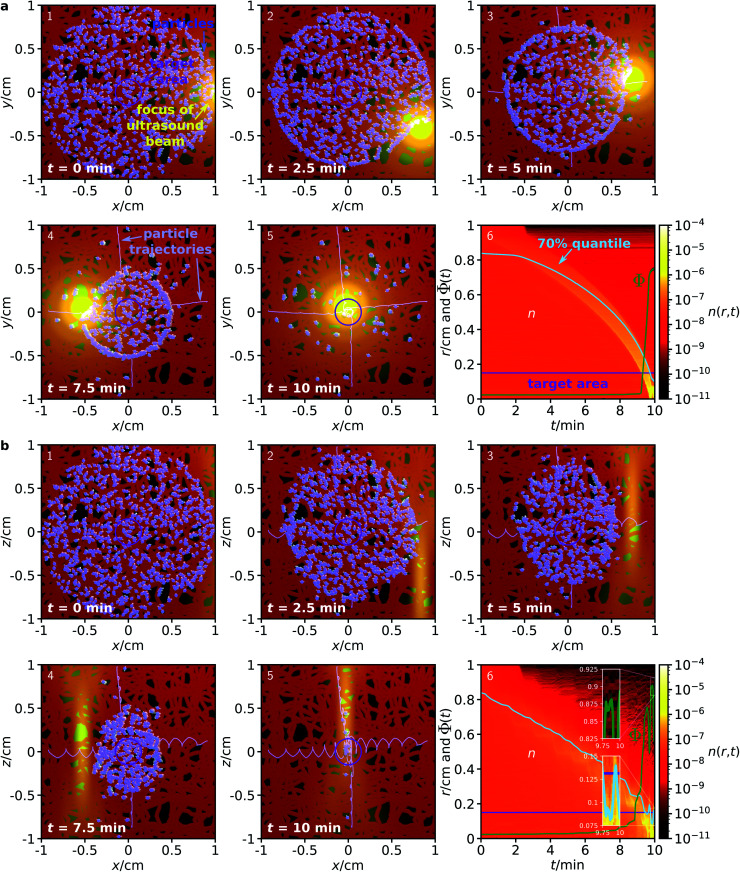
Analogous to Fig. 2, but now for a complex environment given by a network of channels with diameter 500 μm to which the particles are confined.

As can be seen from the results in Fig. 3, the proposed method works surprisingly well even for particles in this very complex environment, although its efficiency is naturally slightly reduced compared to the fully homogeneous case. Due to the confinement, some of the particles get trapped in the network and cannot reach the target. For particles in an ensemble of 100 simulations in the *x*–*y* plane, in average a fraction of 74.76 ± 0.70% of the particles reaches the target within a 10 minute period, while the corresponding fraction for particles in the *x*–*z* plane is 89.28 ± 1.02%. While these figures are already surprisingly good, they could be further improved by repeated sweeps of the two fields. Hold times between two sweeps could potentially improve the results even further. During these hold times, the particles can rearrange (*e.g.*, by diffusion), allowing them to reach a position from where they have higher success in being guided to the target by the next sweep.

## III. Conclusions

In this article, we have proposed and computationally demonstrated a method for the collective guiding of acoustically propelled nano- and microparticles. This method is based on an experimentally realizable combination of a space- and time-dependent acoustic field and a time-dependent magnetic field. Importantly, the method requires neither knowledge about the initial positions and orientations of the particles, nor any particle tracking during the guiding process. Our results clearly show that the method allows for reliable transport of a large number of arbitrarily distributed particles over long distances to a prescribed target area where they accumulate. The method is furthermore biocompatible, and the position, size, and shape of the target area can be freely chosen. We also found that the method is relatively fast, requiring only a few minutes for particle accumulation in realistic situations, scalable with respect to the number of particles, and remains feasible when the particles are in a complex environment of unknown structure.

These features all make the proposed method potentially applicable for controlling the collective dynamics of ultrasound-propelled active particles for future applications in, *e.g.*, materials science and nanomedicine.^22,23^ In materials science, the method could be used to control the behavior of active materials and, *e.g.*, induce the emergence of tailor-made persistent flow fields. Modified by replacing the ultrasound field with a light field, the method can also be used for guiding light-propelled particles.^49,56–58^ In nanomedicine, the proposed method would be particularly useful, since medical applications go along with strict medical safety requirements that cannot be fulfilled by other approaches to particle guiding. The method is fully complementary to and can be synergistically combined with techniques from pharmacy and medicine that have been developed for pharmaceutical applications of nanoparticles (*e.g.*, encapsulation and release of drugs, functionalization, stealth features). For example, nanocarriers for targeted drug delivery, which have been intensively investigated in the last two decades, can lead to the accumulation of therapeutic agents, but a large fraction of the administered nanocarriers reaches off-target regions and causes serious adverse drug reactions.^15^ By combining these particles with an ultrasound-propelled and magnetic unit, they would become motile and could be guided towards their target, significantly increasing the targeting efficiency. Besides particles for drug delivery, other nanoparticles, such as magnetic particles for thermal treatment of the target tissue,^59^ could potentially be combined with our method.

Compared to our proof-of-concept simulations, the performance of the proposed method can be further improved by optimization of the values of its various parameters. For example, the profile and trajectory of the ultrasound beam can be modified. One could consider also more complicated ultrasound fields, as long as they can be realized by conventional transducers or acoustic holography.^60^ The ultrasound-propelled particles provide additional options for modifications. Besides improving the performance of the guiding method for the systems that we address in the present work, the strong adjustability of the guiding method and particles allows to adapt the method to a particular application, such as for particle guiding in a real vascular system.

A related task for future research is to test the proposed guiding method in more complex simulations that are more realistic with regard to an application of ultrasound-propelled particles in targeted drug delivery. By showing that the proposed method works for free particles and for particles that are confined in a channel system, the present work already demonstrates that the guiding method solves crucial problems of previous approaches towards guiding of motile particles. The next steps will be to choose a more realistic channel system whose structure closely resembles an actual human vasculature and to incorporate the effects of blood flow and hydrodynamic interactions on the particle motion. Elaborate models for the vascular system could be obtained from sophisticated computer simulations^61,62^ or experimental data.

It would also be interesting to test the proposed guiding method for systems and parameters relevant for the various other (non-medical) potential applications of ultrasound-propelled particles. An example is to study how the guiding method performs for much smaller or larger particles.

## IV. Methods

The numerical results of this work were obtained by Brownian dynamics simulations, whose details we present in the following.

### A. Setup and parameters

We consider a three-dimensional system that includes *N*_p_ particles, an ultrasound field, and a magnetic field. The system is either free of barriers or contains a channel network system confining the particles. To show that the particle guiding works in all dimensions, we consider a particle distribution in the *x*–*y* plane and a particle distribution in the *x*–*z* plane. Initially, the particles are randomly and homogeneously distributed in a circular region of diameter 2 cm. We simulate *N*_p_ = 1000 particles, which corresponds to an areal packing density of 1.96 × 10^−8^, realistic for drug delivery applications. However, a variation of *N*_p_ should not alter the results significantly. The target is in the center of this region and given by a small circular region of diameter 3 mm. Thus, the particles' distance from the center of the target is up to 1 cm. This is much more than typical distances in the order of 50–100 μm (ref. 21, 27 and 46) over which particle guiding has been demonstrated in experiments so far. The particles are propelled by an ultrasound beam antiparallel to the *z*-axis, and oriented by a magnetic field. Both are described below.

### B. Details about the particles

The maximal particle speed needs to be larger than the speed of the blood flow in the capillaries (about 200 μm s^−1^ (ref. 63) in the smallest capillaries), which constitute by far the largest fraction of the vasculature. For particular acoustically propelled particles, a speed of about 250 μm s^−1^ has been reported.^27^ Since the particle speed depends strongly on the shape^23,24^ and size^21^ of the particles and other parameters (including the frequency of the ultrasound), a strong enhancement of the particle speed by optimization of the parameters can likely be achieved.^23^ Furthermore, the particle speed can be enhanced by increasing the ultrasound intensity, since these quantities are approximately proportional to each other.^23^ In the experiments of ref. 20, ultrasound-propelled rod-shaped particles were observed to have a propulsion speed of up to 200 μm s^−1^. In these experiments, it was also observed that the particles begin to levitate when the particles are exposed to pulsed ultrasound with a duty cycle of 0.04 and a frequency of 3.7 MHz. Equating the gravitational force of 0.027 pN (ref. 20) acting on the particles and the expression for the acoustic radiation force acting on a cylindrical particle^64^ yields an acoustic energy density of 2.20 J m^−3^ that corresponds to the levitation threshold. For their main experiments they used the same input voltage for the ultrasound transducer but continuous ultrasound (corresponding to a duty cycle of 1), *i.e.*, an acoustic energy density of (2.20/0.04) J m^−3^ = 55 J m^−3^. If using the same ultrasound intensity in the focus of our ultrasound beam, the mean ultrasound intensity in the spherical region in which the particles are initially distributed in our simulations would be about 1.22% of the ultrasound intensity in the focus, *i.e.*, about 0.67 J m^−3^. According to the U.S. Food and Drug Administration, intensities of up to 720 mW cm^−2^, which corresponds to 4.8 J m^−3^ (assuming a realistic sound velocity of *c* = 1500 m s^−1^ in tissue^65^), are considered as harmless and therefore allowed for diagnostic ultrasound.^66^ Therefore, the ultrasound intensity could be increased by a factor of about 7, which would enhance the particle speed from 200 μm s^−1^ to 1.4 mm s^−1^. Since already the optimization of the particles' shape and size and an increase of the ultrasound intensity allow for reaching much faster particle speeds than in ref. 20, for our simulations we assume a particle speed of 1 mm s^−1^ in the focus of the ultrasound beam.

Since acoustically propelled particles can have very different shapes and the optimal particle shape still needs to be found,^20,21,55^ in our simulations we make the simple assumption of spherical particles. Furthermore, since the propulsion speed is an independent parameter in our simulations and since particle interactions are almost negligible due to the low packing density of the particles, the assumed particle shape should have no considerable effect on our simulation results.

Similarly, there is no obvious choice of optimal particle size. The lower boundary for the particle size is at about 10 nm, since smaller particles are filtered out by the kidney^12,67^ when they circulate through the vascular system. On the other hand, the particles should be sufficiently small so that they do not clog the capillaries, whose minimal diameter is 5 μm,^15,61^ and cause thromboses.^7^ Furthermore, the particles should be able to pass the liver, which requires a size below 100–150 nm.^67^ Another important advantage of particles that have a size of 100 nm or less is that they can pass the leaky vasculature of tumors and benefit from the enhanced permeability and retention effect, which leads to an accumulation within tumors.^12,67^ Therefore, we chose a particle size of 100 nm for our simulations. With this size, the particles are at the upper end of the size range for nanoparticles and at the lower end of the size range for microparticles.

There are still many options for the particular design of the particles. An example design that is realistic for drug delivery applications is shown in Fig. 1f. It has a magnetized ferrimagnetic core with a diameter of 80 nm and a 10 nm thick shell of a bulk material with embedded therapeutic agents. In addition, the surface of the particle can be functionalized. All materials used in the particle should be biocompatible, *i.e.*, nontoxic and biodegradable. The core of the particle does not need to be massive. It can consist of a large number of magnetic nanoparticles. A suitable material for the core is magnetite.^68–70^ As a particle with a size of about 80 nm, magnetite is ferrimagnetic and has a remanent magnetic moment of *μ* = 1.394 × 10^−17^ J T^−1^. This value follows from a remanent magnetic moment density of about 10 emu g^−1^ (ref. 71) and a mass density of 5.2 g cm^−3^.^72^ The magnetization of the core allows to align the particle orientation by a homogeneous magnetic field. For convenience, we choose the same orientation for the particle, its propulsion, and its magnetization. The material of the shell should degrade slowly when it is in contact with biofluids. For this purpose, one could choose Al, Ca, Mg, Si, or Zn or chemical compounds based on them, such as calcium carbonate, calcium sulphate, and calcium phosphate.^19,43,73^

A surface functionalization of the particles would allow to improve the retention in the body, by equipping the particles with stealth features, and thus the success of a therapy.^10^ This can also help to control the speed of degradation of the particles. For this purpose, the functionalization techniques that have been developed in pharmacy and medicine can be utilized. An option is to cover the particles by a suitable lipid (bi)layer (*e.g.*, consisting of phospholipids), proteins (*e.g.*, human serum albumin or gelatin), or hydroxyethyl starch.

### C. Details of the ultrasound field

The focused ultrasound beam can be generated by a phased array transducer^74^ or a transducer consisting of an oscillating spherical cap.^75,76^ We consider here the second case in more detail, since it is relatively simple to realize. The ultrasound field can be calculated by solving the wave equation. For the acoustic pressure amplitude *p* at position *r⃑* = (*x*,*y*,*z*)^T^ around a fixed transducer, one obtains the Rayleigh–Sommerfeld integral^76^1
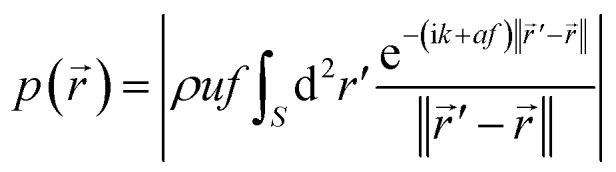
with the absolute value |·|, mass density of the surrounding medium (tissue) *ρ*, velocity amplitude of the oscillating cap *u*, and frequency of the ultrasound *f*. The integral is performed over the spherical-cap-shaped surface *S* of the transducer, i is the imaginary unit, *k* = 2π*f*/*c* is the wave number of the ultrasound, *c* is the sound velocity in tissue, *a* = 1 dB cm^−1^ MHz^−1^ = 5 ln(10) m^−1^ MHz^−1^ is the attenuation coefficient in tissue,^65^ and ‖·‖ denotes the Euclidean norm. We assume that the radius of curvature and the diameter of the spherical-cap-shaped transducer are 20 cm.

The propulsion speed of the particles is approximately proportional to the sound intensity *I* ∝ *p*^2^.^23^ Since we prescribe the propulsion speed of the particles in the center of the focus of the ultrasound beam by an independent parameter (see Section IVB), we are only interested in the spatial profile of *p*^2^(*r⃑*), but not on the overall prefactor in eqn (1). Therefore, we normalize the maximum of *p*(*r⃑*) to 1 and do not need to assume particular values for *ρ* and *u*. For the ultrasound frequency, we choose a value of *f* = 1 MHz, since this value lies in the frequency range of therapeutically used ultrasound^75^ and leads to a good compromise between high efficiency and high resolution of the particle guiding. Smaller frequencies would have the advantage that they lead to a wider focus of the ultrasound beam so that more particles are propelled at the same time, but they would also reduce the spatial resolution for the particle guiding. As a realistic value for the sound velocity in tissue, we choose *c* = 1500 m s^−1^.^65^

In a simple setup, the focus of the ultrasound beam is moved by moving the transducer. For convenience, we assume that the ultrasound beam is always antiparallel to the *z*-axis but can be moved in all three directions. In contrast to the intensity profile for a phased array transducer, the profile of a spherical-cap-shaped transducer has a constant structure and is just shifted as a function of time. For the latter case, the intensity profile has an axis of rotational symmetry and its full-width-at-half-maximum shape is similar to a prolate spheroid with dimensions 1.585 mm and 10.107 mm. The normalized intensity profile can approximately be represented analytically by the function2
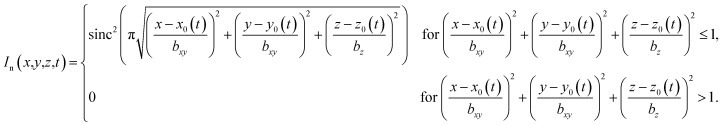
Here, *x*_0_(*t*), *y*_0_(*t*), and *z*_0_(*t*) are the time-dependent coordinates of the center of the focus of the ultrasound beam and *b*_*xy*_ = 1.790 mm and *b*_*z*_ = 11.408 mm are fit parameters that determine the aspect ratio *χ* = *b*_*z*_/*b*_*xy*_ ≈ 6.38 of the spheroid.

### D. Details of the magnetic field

For the magnetized particles described in Section IVB, a homogeneous magnetic field is sufficient to align them in any wanted direction. Such a magnetic field could be generated, *e.g.*, by a pair of Helmholtz field coils. To reorient the magnetic field, one could use a pair of coils that can be rotated around its center or three pairs of static and pairwise perpendicular field coils whose flux densities can be tuned independently. For the magnetic field, we use a constant flux density of 1 mT. Since the magnetic field rotates with the orbital frequency of the ultrasound focus, which is below 0.1 Hz, its flux density is harmless. Taking into account that the extension of the magnetic field is about 2 m in a realistic application, the electric field in a human body that can be induced by the rotation stays below 5 × 10^−4^ V m^−1^, which does not cause health damages.^77^ Rotating magnetic fields with a similar strength are also used in other research projects on medical applications.^39,40,42^

### E. Details on the simulations

The simulations were performed using the software package Lammps.^78^

#### 1. Equations of motion

We consider a system of *N*_p_ = 1000 spherical particles with diameter *σ* = 100 nm in two spatial dimensions, where the target is in the origin of coordinates. The equations of motion of the particles are the overdamped Langevin equations^1^3
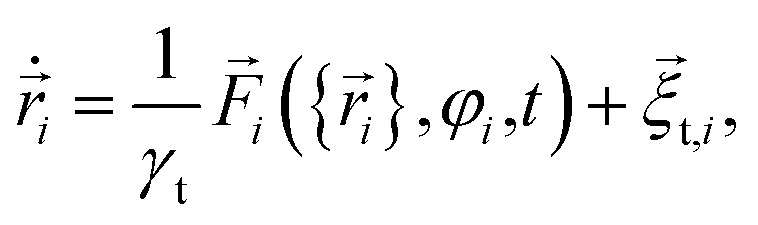
4
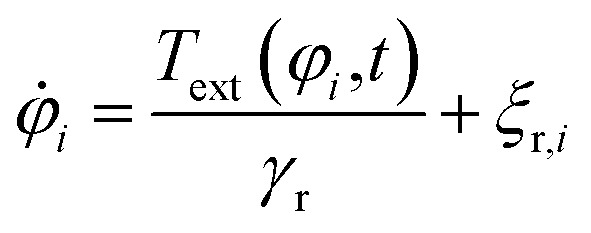
that describe the position *r⃑*_*i*_(*t*) and orientation *φ*_*i*_(*t*) of the *i*-th particle as functions of time *t*. Since the particles are spheres, their translational and rotational friction coefficients are *γ*_t_ = 3π*ησ* and *γ*_r_ = π*ησ*^3^, respectively, with the shear viscosity of blood plasma *η* = 10^−3^ Pa s.^79^*F⃑*_*i*_ is the total force acting on particle *i*, *T*_ext_ is the external torque which the magnetic field exerts on the particles, and *ξ⃗*_t,*i*_ and *ξ*_r,*i*_ are zero-mean Gaussian white noise terms that take Brownian motion of particle *i* into account.

The force *F⃑*_*i*_ is given by

5 *F⃑*_*i*_({*r⃑*_*i*_},*φ*_*i*_,*t*) = *F⃑*_int,*i*_({*r⃑*_*i*_}) + *F⃑*_p_(*r⃑*_*i*_,*φ*_*i*_,*t*) + *F⃑*_ext_(*r⃑*_*i*_).

Its first contribution is the interaction force6
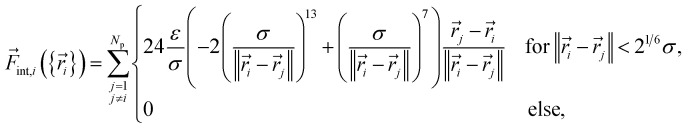
which is based on the assumption that the particles interact by the Weeks–Chandler–Andersen potential^80^7
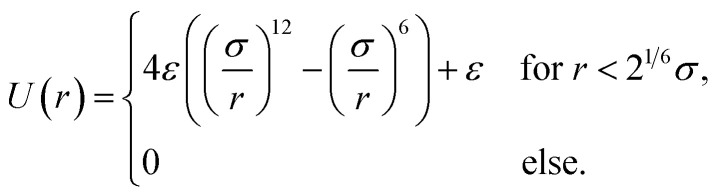
Here, *ε* is the characteristic interaction energy. It is the potential energy of a pair of particles with distance *r* = *σ* and here chosen as *ε* = *k*_B_*T*_b_ ≈ 4.28 × 10^−21^ J with the Boltzmann constant *k*_B_ and the body temperature *T*_b_ = 37 °C ≈ 310 K. This choice ensures that nondriven particles that move only by Brownian motion keep a typical distance of *σ* from each other. We do not take magnetic interactions of the particles into account, since the packing density of the particles is very low, the magnetic interactions would strongly depend on assumptions about the particle design, and for particles with a small magnetic core the magnetic interactions can be relatively small.

The second contribution to eqn (5) is the acoustic propulsion force *F⃑*_p_ that acts on a particle. It is given by

8 *F⃑*_p_(*r⃑*,*φ*,*t*) = *v*_0_*γ*_t_*I*_n_(*r⃑*,*t*)*û*(*φ*)

with the propulsion speed *v*_0_ = 1 mm s^−1^ that we prescribe in the center of the focus and the orientational unit vector *û*(*φ*) = (cos(*φ*),sin(*φ*))^T^ corresponding to the orientation angle *φ*. The third contribution to eqn (5) is the external force *F⃑*_ext_. It is zero when we consider a system without boundaries, but important in our simulations with the channel system. In the latter case, *F⃑*_ext_(*r⃑*) is the force that the channel walls exert on a particle at position *r⃑*. For the interactions of a particle with the channel walls, we use again the Weeks–Chandler–Andersen potential (7).

The external torque *T*_ext_(*φ*,*t*) in eqn (4), which the external magnetic field exerts on a particle with orientation *φ*, is given by

9 *T*_ext_(*φ*,*t*) = (*

<svg xmlns="http://www.w3.org/2000/svg" version="1.0" width="13.000000pt" height="16.000000pt" viewBox="0 0 13.000000 16.000000" preserveAspectRatio="xMidYMid meet"><metadata>
Created by potrace 1.16, written by Peter Selinger 2001-2019
</metadata><g transform="translate(1.000000,15.000000) scale(0.012500,-0.012500)" fill="currentColor" stroke="none"><path d="M640 1080 l0 -40 -160 0 -160 0 0 -40 0 -40 160 0 160 0 0 -40 0 -40 40 0 40 0 0 40 0 40 40 0 40 0 0 40 0 40 -40 0 -40 0 0 40 0 40 -40 0 -40 0 0 -40z M320 720 l0 -80 -40 0 -40 0 0 -120 0 -120 -40 0 -40 0 0 -120 0 -120 -40 0 -40 0 0 -80 0 -80 40 0 40 0 0 80 0 80 40 0 40 0 0 40 0 40 120 0 120 0 0 40 0 40 40 0 40 0 0 -40 0 -40 40 0 40 0 0 40 0 40 40 0 40 0 0 40 0 40 -40 0 -40 0 0 -40 0 -40 -40 0 -40 0 0 80 0 80 40 0 40 0 0 120 0 120 40 0 40 0 0 40 0 40 -40 0 -40 0 0 -40 0 -40 -40 0 -40 0 0 -120 0 -120 -40 0 -40 0 0 -80 0 -80 -120 0 -120 0 0 40 0 40 40 0 40 0 0 120 0 120 40 0 40 0 0 80 0 80 -40 0 -40 0 0 -80z"/></g></svg>

*(*φ*) × *B⃑*(*t*))·*ê*_*z*_.

Here, **(*φ*) = *μû*(*φ*) is the vectorial and *μ* = 1.394 × 10^−17^ J T^−1^ the scalar magnetic moment of a particle with orientation *φ*, × denotes the cross product,10
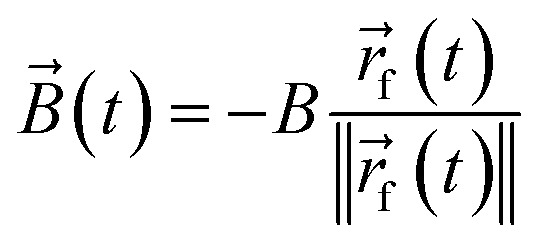
is the vectorial flux density of the time-dependent magnetic field, and *ê*_*z*_ is the unit vector in the *z* direction. *r⃑*_f_(*t*) is the position of the focus of the ultrasound beam at time *t*. The absolute flux density *B* = ‖*B⃑*‖ = 1 mT of the magnetic field is sufficiently low to be harmless in medical applications but large enough to cause an alignment of the particles' orientation.

Finally, the zero-mean Gaussian white noises *ξ⃗*_t,*i*_(*t*) and *ξ*_r,*i*_(*t*) in eqn (3) and (4) are statistically independent. *ξ⃗*_t,*i*_(*t*) describes translational Brownian motion and *ξ*_r,*i*_(*t*) describes rotational Brownian motion. Their correlation is given by

11 〈*ξ⃗*_t,*i*_(*t*)⊗*ξ⃗*_t,*j*_(*t*′)〉 = 2*D*_t_*δ*_*ij*_*δ*(*t* − *t*′)**1**,

12 〈*ξ*_r,*i*_(*t*)*ξ*_r,*j*_(*t*′)〉 = 2*D*_r_*δ*_*ij*_*δ*(*t* − *t*′)

with the ensemble average 〈·〉, dyadic product ⊗, translational diffusion constant *D*_t_ = *k*_B_*T*_b_/*γ*_t_, rotational diffusion constant *D*_r_ = *k*_B_*T*_b_/*γ*_r_, Kronecker delta *δ*_*ij*_, delta distribution *δ*(*t*), and unit matrix **1**.

The equations of motion (3) and (4) are solved by the Euler–Maruyama integration scheme. We arranged the simulations to stop if a particle leaves the simulation domain. However, this case did not occur in our simulations. We used a time step size Δ*t* = 0.3 μs, which is found to be needed to resolve the particle interaction correctly. We simulated a period of *t*_s_ = 10 min, which proved to be sufficient for guiding the particles to the target.

#### 2. Trajectory of the ultrasound beam

The focus of the ultrasound beam moves along a spiral towards the center of the target, which is in the origin of coordinates. For the simulations in the *x*–*y* plane, the spiral is circular, and for the simulations in the *x*–*z* plane, it is ellipsoidal. We chose the distance of neighboring revolutions equal to the focus diameter. When the edge of the focus touches the center of the target, the ultrasound beam starts to move one last time around the center. In this final orbit, the edge of the focus permanently touches the center of the target. This last orbit increases the fraction of particles that reach the target.

For the *x*–*y* system, the trajectory of the focus is given by

13 *r⃑*_f_(*t*) = *r*_f_(*t*)*û*(*φ*_f_(*t*))

with the focus position *r⃑*_f_(*t*) = (*x*_f_(*t*),*y*_f_(*t*))^T^, the distance of the center of the focus from the origin of coordinates

14 *r*_f_(*t*) = *R*_f_ + *R*_0_(1 − *s*(*t*)),

and the angle15
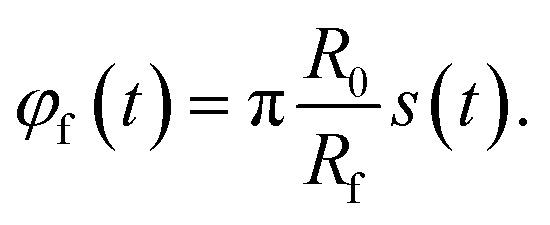
Here, *R*_f_ = 0.7927 mm is the radius of the focus in the *x*–*y* plane, *R*_0_ = 1 cm is the radius of the region in which the particles are initially distributed, and16
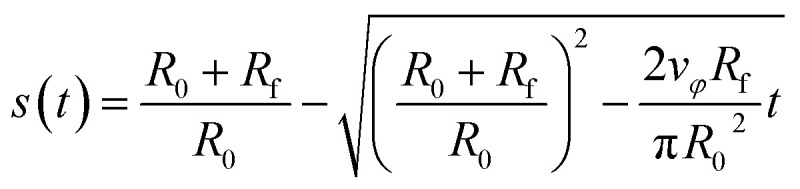
with *s*(*t*) ∈ [0, 1] is the trajectory parameter. The constant *v*_*φ*_ = 0.391 mm s^−1^ is the tangential speed with which the focus moves around the center of the target. Its value is chosen so that the duration of a guiding run is *t*_s_ = 10 min. At time17
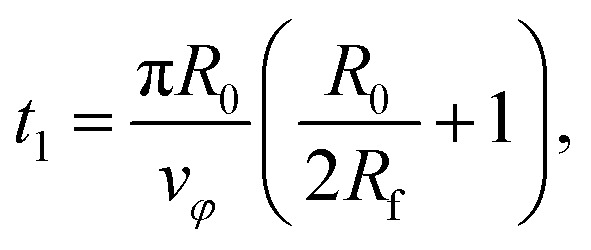
*s* is equal to 1 and the focus touches the center. Then, the final circle of the trajectory starts. It has radius *R*_f_ and takes a period of18
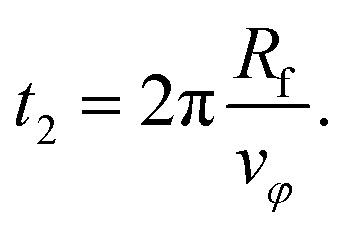


The total duration of a simulation run is therefore given by *t*_s_ = *t*_1_ + *t*_2_.

For the *x*–*z* system, the trajectory of the focus is, instead of eqn (13), given by the focus position *r⃑*_f_(*t*) = (*x*_f_(*t*),*z*_f_(*t*))^T^ with

19 *r⃑*_f_(*t*) = *r*_f_(*t*)*û*(*φ*_f_(*t*)) + (*χ* − 1)*R*_f_ sin(*φ*_f_(*t*))*ê*_*z*_,

where *χ* = 6.38 is the aspect ratio of the spheroidal focus region. Now, *r*_f_(*t*) can no longer be interpreted as the distance of the center of the focus from the origin of coordinates, *v*_*φ*_ is no longer the tangential speed of the focus, and the final orbit is an ellipse with aspect ratio *χ*. It is described by eqn (19) with *r*_f_ = *R*_f_ and *φ*_f_(*t*) = π*R*_0_/*R*_f_ + (*v*_*φ*_/*R*_f_)(*t* − *t*_1_).

#### 3. Channel system

Here, we describe the algorithm that we used to generate the channel systems. Each channel has width *w* = 500 μm (ref. 81) and consists of two walls separated by distance *w*. The walls are realized by a chain of small spherical wall particles. These particles have fixed positions, the same diameter of 100 nm as the regular particles, and a center-to-center distance of 50 nm from each other. The interaction of an ultrasound-propelled particle with a wall particle is the same as the regular particle–particle interaction given by eqn (6). This interaction causes a confinement of the particles in the channel system.

The channels are created by discrete random walks in a quadratic domain of size *l*_qd_ = 2.4 cm with step size *w*. This domain is slightly larger than the domain of size *l* = 2 cm in which the particles are distributed, to avoid boundary effects of the channel algorithm and to have a safety distance between the particles and the boundaries of the simulation domain. Each random walk starts at the boundary of the domain with a step towards the interior of the domain. All steps are made in random directions that deviate by an angle Δ*α* from the direction of the previous step, where the reference orientation of the initial step is perpendicular to the domain boundary. This random angle follows the probability distribution20
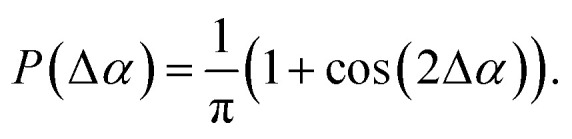


To increase the persistence length of the random walk and thus to avoid loops of the random paths, the angle *α* is restricted to *α* ∈ [ −π/4, π/4]. When a random walk reaches the boundary of the domain again, it is stopped. The starting points of the random walks are equally spaced with distance *d* = 1 mm along the full boundary of the domain.

To create the channel system from the random walks, spheres with diameter *w* are placed at each point of the random paths. Furthermore, cylinders with diameter *w* and length *w* are placed along the steps of the random walks such that the centers of their upper and lower bases coincide with two subsequent points of a random walk. Finally, the spheres and cylinders are merged, which yields a smooth channel system. The walls of the channel system are given by its surface within the domain.

**Fig. 4:**
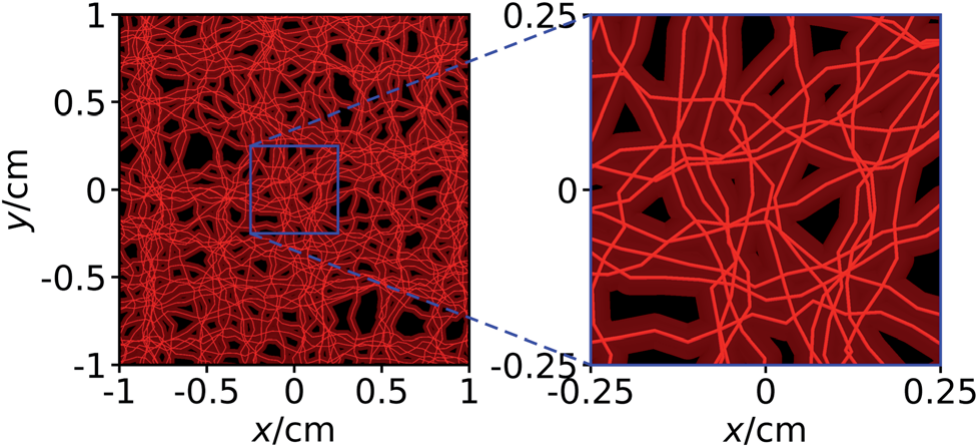
The same channel system as shown in Fig. 3. In addition to the channels, we here show the axes of the channels that form a network.

For each simulation run, an individual channel system is generated so that our simulation results that originate from an averaging over simulation runs correspond to averaging over different initial particle distributions and different channel systems. As an example, Fig. 4 shows one of the channel systems that we used in our simulations and that corresponds to Fig. 3.

## Conflicts of interest

There are no conflicts of interest to declare.

## Supplementary Material
